# Research progress on the role and mechanisms of ferroptosis in diabetic wound repair

**DOI:** 10.1038/s41420-025-02808-y

**Published:** 2025-11-07

**Authors:** Wei Zhang, Heng He, Shaofeng Chen, Sujie Xie, Xirui Tong, Xinran Ding, Yixu Li, Shuyuan Xian, Runzhi Huang, Zhaofan Xia, Shizhao Ji

**Affiliations:** 1 Department of Burn Surgery, The First Affiliated Hospital of Naval Medical University, Shanghai, China; 2 Department of Orthopaedic Surgery, China Coast Guard Hospital, Zhejiang, China; 3 Research Unit of key techniques for treatment of burns and combined burns and trauma injury, Chinese Academy of Medical Sciences, Shanghai, China

**Keywords:** Endocrine system and metabolic diseases, Metabolic disorders

## Abstract

Diabetic wounds remain a formidable clinical challenge due to their delayed healing, frequent infections, and high recurrence rates. Ferroptosis, an iron-dependent form of regulated cell death driven by lipid peroxidation, may hinder diabetic wound repair through multifaceted mechanisms. This review elucidates the regulatory pathways of ferroptosis, focusing on its disruptive effects on critical reparative cells (macrophages, fibroblasts, endothelial cells, and keratinocytes) and bacterial infections in the wound microenvironment. We further systematically evaluate the therapeutic potential of ferroptosis-targeting agents in promoting diabetic wound healing, thereby providing a theoretical framework for developing precision interventions against ferroptosis.

## Facts

- The refractory healing of diabetic wounds poses significant clinical challenges, with ferroptosis playing a critical role in the pathological repair process of these wounds.
- The pathological microenvironment of diabetic wounds induces abnormal regulation of ferroptosis in multiple repair cells, disrupting the normal process of wound repair.
- Therapeutic strategies targeting cellular ferroptosis hold promise for improving cellular function and promoting the healing of diabetic wounds.

## Open questions

- How does ferroptosis in distinct cell types within the diabetic wound microenvironment (e.g., endothelial, fibroblastic, macrophage, and keratinocyte cells) influence the wound repair process?
- What are the key molecular mechanisms underlying the dysregulation of ferroptosis in wound repair cells under diabetic conditions?
- What drugs and intervention modalities have been developed thus far for therapies based on ferroptosis?

## Introduction

Diabetic wounds represent one of the common and severe complications in diabetic patients, characterized by high incidence and difficult healing, with diabetic foot ulcer (DFU) serving as a typical exemplar. Diabetes poses a global health challenge. Statistics indicate that approximately 537 million individuals worldwide are currently living with diabetes [1], among whom 19–34% will develop DFU [2, 3]. With the annual increase in diabetes prevalence, the number of diabetic patients is projected to reach 1.31 billion by 2050 [4], imposing a heavy burden on both patients and healthcare systems. Additionally, diabetic wounds are prone to severe infections. Approximately 50%-60% of ulcerative wounds become infected, with 20% of moderate-to-severe infections ultimately leading to lower limb amputation [3]. Meanwhile, due to the presence of vascular lesions, neurological dysfunction, and repeated treatment failures in ulcerative wounds, DFU have also emerged as a primary cause of non-traumatic lower limb amputation [5]. Current standard clinical treatments for DFU include surgical debridement, decompression therapy, vascular reconstruction, infection control, and dressing applications [6]. However, only 30% of patients achieve wound healing after 20 weeks of standardized treatment [7]. The 5-year mortality rate among DFU patients is ~30%, while the 5-year mortality rate for patients undergoing major amputation is more than 70% [3]. Concurrently, DFU exhibit a high recurrence rate, with up to 42% recurrence within one year [3]. These findings highlight the critical need to decipher the molecular mechanisms underlying impaired diabetic wound healing and develop targeted effective therapeutic agents. Such efforts are pivotal for promoting wound healing in diabetic patients and alleviating the disease burden associated with diabetic wounds.

Wound healing requires a series of coordinated processes, with each phase involving multiple cell types [8]. The initiation, maintenance, and completion of the repair process are critical for successful wound closure [9]. However, in diabetic patients, wound healing is often disrupted by multiple factors, causing dysregulation in hemostasis, inflammation, proliferation, and remodeling phases, which ultimately lead to chronic wounds. Moreover, abnormal wound healing lays a foundation for various chronic diseases, including inflammatory disorders, fibrosis, and cancer [10]. Compared with acute wounds, DFU are characterized by persistent chronic inflammation, impaired angiogenesis, stagnant re-epithelialization, and difficult remodeling [11, 12]. Meanwhile, under the harsh environment of long-term high blood glucose levels, accumulation of advanced glycation end products (AGEs) and high levels of reactive oxygen species (ROS) and inflammatory factors in diabetic wounds, the relevant cells involved in wound repair will be functionally impaired or even die, which seriously affects the normal healing process of wounds [11].

Ferroptosis is a form of regulated cell death (RCD) triggered by specific perturbations in the intracellular microenvironment, particularly severe lipid peroxidation and iron overload [13–16]. The concept of ‘ferroptosis’ was first proposed by Dixon in 2012, and is defined as a non-apoptotic form of RCD because its mechanism is independent of the cysteine aspartic proteases that are involved in apoptosis [17–19]. Notably, ferroptosis is iron-dependent and differs morphologically and biochemically from other types of RCD. Morphologically, ferroptosis primarily exhibits a necrotic phenotype, with mitochondrial alterations being dominant, such as significant mitochondrial shrinkage, increased membrane density, and reduction or loss of mitochondrial cristae and outer membranes [20]. Biochemically, ferroptosis mainly involves iron overload, lipid peroxidation, Fenton reactions, and cystine deprivation [21]. Ferroptosis has been reported to widely participate in the pathogenesis of many common diseases, including ischemia-reperfusion injury, inflammatory bowel disease, liver fibrosis, acute kidney injury, stroke, Alzheimer’s disease, cardiovascular diseases, immunity-related disorders, and cancer [22]. Additionally, its role in diabetes and its complications, such as diabetic nephropathy, retinopathy, cardiomyopathy, and microangiopathy, has been intensively studied [23]. A prospective clinical study has demonstrated that ferroptosis levels showed considerable promise as a novel monitoring indicator and therapeutic target, as they play a critical role in predicting the healing rate of DFUs and assisting clinical treatment decisions [24]. As a common complication of diabetes, diabetic wounds have garnered increasing research attention in recent years, primarily focusing on cell types involved in wound repair, such as macrophages, fibroblasts, endothelial cells, and keratinocytes.

Understanding the interplay between diabetic wounds and ferroptosis holds potential significance for developing novel pharmacological strategies. Therefore, this review summarizes the mechanisms of ferroptosis, its functional impacts on wound repair-related cells (macrophages, fibroblasts, endothelial cells, keratinocytes) and wound bacterial infections. Additionally, we further outline potential therapeutic strategies, aiming to provide research insights for developing new targets and technologies in the treatment of diabetic wounds.

## Healing characteristics of diabetic wounds

### Normal wound repair process

Wound healing is a dynamically regulated process composed of multi-stage biological events, encompassing core processes such as necrotic tissue clearance, granulation tissue formation, and epidermal barrier function reconstruction. Its mechanism relies on spatiotemporal coordination among epidermal cells, immune cells, and fibroblasts through extracellular matrix (ECM) remodeling and signaling factor cascades, involving multi-dimensional molecular interaction networks such as gene expression regulation, metabolic reprogramming, and microenvironmental homeostasis. Current evidence indicates that wound healing can be divided into four phases: hemostasis, inflammation, proliferation, and remodeling [8].

During the hemostasis phase, fibrinogen is exposed, triggering platelet adhesion and activation with the release of multiple factors [25]. Subsequently, the coagulation system is activated, converting fibrinogen into an insoluble fibrin network to form blood clots. Ultimately, damaged blood vessels are sealed, and bleeding ceases. In the inflammation phase, neutrophils and macrophages serve as core effector cells, recruited to the wound injury microenvironment via chemokine-mediated directional migration [26]. Their functions include pathogen phagocytosis, necrotic debris degradation, and time-ordered release of pro-healing factors (e.g., VEGF, PDGF), thereby regulating the transition from inflammation to repair homeostasis. The proliferation phase represents a critical repair stage with balanced immune and inflammatory responses. Multiple cell types, including fibroblasts, endothelial cells, and keratinocytes, participate in wound repair by forming new tissues to fill defects, such as neovessels, ECM components, and granulation tissue, ultimately achieving epithelialization [27–29]. In the final remodeling phase, the ECM undergoes coordinated dynamic processes of synthesis and degradation, which is accompanied by increasing of ECM tensile strength and reduction in blood supply to the injured tissue bed [30, 31]. Clinically, imbalance in this phase leads to poor wound healing, potentially resulting in weak wound strength or the formation of hypertrophic scars or keloids.

### Delayed healing of diabetic wounds

Optimal wound healing requires the fine integration of complex biological processes and molecular events, such as cell migration, proliferation, ECM deposition, and remodeling. However, the orderly progression of this healing process is impaired in chronic wounds, represented by diabetic wounds [32]. Studies indicate that chronic wounds like DFU do not follow an orderly healing sequence and may stagnate at different repair stages, leading to delayed healing [33, 34]. The mechanisms underlying abnormal wound healing in diabetes are complex and diverse, primarily manifested in hyperglycemia-induced oxidative stress, inflammatory responses, and significant impairment of cellular functions.

During the hemostasis phase, abnormal platelet accumulation exacerbates local vascular damage [35]. In the inflammation phase, inflammatory cells are critical for clearing necrotic tissue and resisting pathogenic microbial invasion. In diabetic patients, non-healing wounds typically exhibit impaired inflammatory responses in the early stage after injury, followed by a delayed exacerbation of the inflammatory state [36]. For example, reduced expression of inflammatory cytokines in monocytes or macrophages has been observed in the early stage of diabetic wound injury [37]. Analyses based on single-cell RNA sequencing and spatial transcriptome data have also revealed higher abundances of M1-type macrophages in the wound skin tissues of healed DFU patients, while non-healed patients show higher abundances of M2-type macrophages [38]. However, the observed high abundance of M1-type macrophages in healed DFU patients does not contradict the widely accepted view that impaired M2 polarization of macrophages contributes to poor healing of diabetic wounds [39, 40]. This apparent discrepancy requires comprehensive consideration of the stage and progression of wound repair. During the early stage of repair, inflammatory activation helps clear pathogens and damaged tissue, facilitating the transition from the inflammatory phase to the proliferative phase, thereby promoting the healing process of DFU wounds [38, 41, 42]. In the later stages of wound repair, M2 polarization plays a critical role by producing anti-inflammatory, fibrogenic, and angiogenic mediators to limit local inflammation and promote tissue repair [43, 44]. In the proliferation phase, the proliferation, migration, and secretory functions of fibroblasts, endothelial cells, keratinocytes, and other cells are crucial for wound closure. However, prolonged oxidative stress and inflammation impair the functions of these cells, leading to insufficient ECM deposition, angiogenesis disorders, delayed epithelialization, and other issues that affect wound healing [45–47]. Additionally, increased intracellular ROS levels and accumulation of lipid peroxides threaten cell survival and induce ferroptosis [48, 49]. Abnormalities of these cells during the remodeling phase may also cause defects in collagen synthesis and reconstruction, further affecting wound healing.

## Mechanisms related to ferroptosis

Ferroptosis is a RCD induced by iron-dependent lipid peroxidation, with distinct signaling regulatory mechanisms compared to other cell death modes (such as apoptosis, necrosis, autophagy, and pyroptosis) [21]. Studies have confirmed that the molecular regulatory network of ferroptosis is highly complex, with its molecular basis involving the synergistic/antagonistic effects of multiple signaling pathways and regulatory factors, which are primarily closely associated with iron overload, lipid peroxidation, and imbalances in the antioxidant system [21, 50]. In this section, we systematically summarize the critical roles of these three mechanisms in ferroptosis.

### Iron overload

As an iron-dependent form of RCD, iron metabolism plays a core role in the occurrence of ferroptosis. Iron overload caused by abnormal iron metabolism is a key inducer of ferroptosis. Iron exists in two forms in the body, Fe²⁺ and Fe³⁺. Under normal conditions, their contents and distributions are in dynamic equilibrium, which is vital for cell survival [51]. Disruption of this dynamic equilibrium may lead to the occurrence of some pathological conditions [52]. Fe²⁺ generated from intestinal absorption and red blood cell degradation are two major sources of iron in the body. Fe²⁺ is first oxidized to Fe³⁺, which then binds to transferrin and is endocytosed by cells. Following endocytosis, Fe³⁺ is reduced to Fe²⁺ by the six-transmembrane epithelial antigen of the prostate 3 (STEAP3), and subsequently stored in mitochondria, the endoplasmic reticulum, ferritin, and the iron pool. Once iron metabolism is disordered, Fe²⁺ stored in aforementioned locations is released, and the iron pool is converted into a labile iron pool, leading to excessive Fe²⁺ content in the cytoplasm [53]. Subsequently, supraphysiological doses of Fe²⁺ can catalyze lipid peroxidation by promoting Fenton reactions or activating iron-dependent enzymes, thereby facilitating ferroptosis [54]. Studies have shown that the iron chelator deferoxamine and the ferroptosis inhibitor Ferrostatin-1 can significantly inhibit ferroptosis and alleviate ferroptosis-related diseases [55, 56].

### Lipid peroxidation

Lipid peroxidation is a critical factor in the occurrence of ferroptosis. Phosphatidylethanolamine-containing polyunsaturated fatty acids (PUFAs), such as arachidonic acid and adrenic acid, are the main substrates for lipid peroxidation [57]. They must be esterified into membrane phospholipids and oxidized to transmit ferroptosis signals, a process dependent on acyl-CoA synthetase long-chain family member 4 (ACSL4) and lysophosphatidylcholine acyltransferase 3 [58–60]. Lipid peroxidation is a free radical chain reaction, mediated by lipoxygenases such as arachidonate 15-lipoxygenase, which recognizes the double-bond sites of PUFAs in membrane phospholipids and converts them into lipid hydroperoxides [57, 61]. Moreover, ROS can also abstract hydrogen atoms adjacent to the double bonds of PUFAs to form lipid radicals, which then combine with oxygen to generate lipid peroxyl radicals, further triggering chain reactions [62, 63]. The ferroptosis inhibitor Ferrostatin-1 can block this chain reaction by capturing lipid radicals [19]. When the antioxidant system is reduced or imbalanced, a large accumulation of lipid peroxides occurs, thereby driving the occurrence of ferroptosis.

### Antioxidant System

Current studies indicate that three major antioxidant defense systems against ferroptosis have been identified: the glutathione peroxidase 4 (GPX4)–glutathione (GSH) system, the ferroptosis suppressor protein 1 (FSP1)–coenzyme Q10 (CoQ10) system, and the dihydroorotate dehydrogenase (DHODH)–coenzyme Q10H₂ (CoQH₂) system. The GPX4-GSH antioxidant system represents the core mechanism by which cells defend against lipid peroxidation and ferroptosis. As a selenium-dependent enzyme, GPX4 specifically catalyzes the reduction of phospholipid hydroperoxides by reduced GSH, converting toxic lipid peroxides into non-toxic lipid alcohols to maintain plasma membrane integrity [64, 65]. Additionally, multiple studies show that cells with downregulated GPX4 expression are more sensitive to ferroptosis compared to control cells [65]. GSH, an essential cofactor for GPX4 activity, is synthesized in a process dependent on the cystine/glutamate antiporter (System Xc⁻). This transporter is a critical amino acid antiporter composed of two subunits: solute carrier family 7 member 11 (SLC7A11) and solute carrier family 3 member 2 (SLC3A2). Extracellular cystine is exchanged with intracellular glutamate through this system, converted to cysteine, and thus maintains intracellular GSH synthesis [19, 65, 66]. Impaired System Xc⁻ function (e.g., inhibition by Erastin) or loss of GPX4 activity (e.g., direct inhibition by RSL3) leads to intracellular GSH depletion and lipid peroxide accumulation, triggering ferroptosis [67, 68]. Additionally, studies revealed that FSP1 can resist ferroptosis independently of GPX4 through the CoQ10 antioxidant system [69, 70]. As an NAD(P)H-dependent ubiquinone reductase localized to the cell membrane, FSP1 reduces CoQ10 to CoQH₂ using NAD(P)H. CoQH₂ scavenges lipid radicals, blocks lipid peroxidation chain reactions, and inhibits ferroptosis. A 2021 study further identified a mitochondrial DHODH-mediated ferroptosis defense mechanism independent of both GPX4 and FSP1 [71]. As an inner mitochondrial membrane enzyme primarily involved in pyrimidine synthesis, DHODH reduces CoQ10 to the specific antioxidant molecule CoQH₂ via the electron transport chain [72].

## Effects of ferroptosis on diabetic wound healing

Impaired repair of diabetic wounds involves dysfunction of multiple cell types, such as macrophages, fibroblasts, endothelial cells, and keratinocytes. Pathophysiological studies indicate that the mechanisms underlying delayed healing of diabetic wounds are multifaceted, with ROS burst and oxidative stress cascades triggered by the high-glucose microenvironment, inducing RCD, which may represent a core cause of non-healing wounds. As a newly identified form of cell death, ferroptosis has been widely reported in recent years to participate in the repair process of diabetic wounds. This section comprehensively describes the functional impacts of ferroptosis on various cell types in diabetic wounds, aiming to provide new insights into the mechanisms of impaired wound healing. Notably, diabetic wounds are prone to infection, and current regulatory strategies targeting pathogen ferroptosis have also attracted extensive attention from researchers, potentially representing a novel approach for treating diabetic wound infections.

### Ferroptosis in macrophages

Macrophages are key players in wound healing, responsible for regulating inflammation, clearing cell debris, and coordinating tissue repair [29]. Diabetic wounds exhibit persistent low-grade chronic inflammation, leading to delayed healing, which may be associated with macrophage dysfunction. Iron levels influence macrophage polarization phenotype switching. Appropriate iron levels promote M2 polarization of macrophages, inducing secretion of cytokines CCL17 and CCL22 that enhance ECM secretion [73], critical for increasing ECM deposition and promoting wound repair [74]. Conversely, iron overload induces M1 polarization by increasing ROS production and promoting p53 acetylation in macrophages, exacerbating inflammatory responses [75]. Macrophages in diabetic environments show distinct morphological features of ferroptosis and altered expression of related molecules, leading to persistent and aggravated inflammation [76]. Treatment with the ferroptosis inhibitor Ferrostatin-1 effectively alleviates high-glucose-induced macrophage injury and ferroptosis, reduces inflammatory responses in diabetic rat wounds, and improves angiogenesis, potentially via upregulating nuclear factor-E2-related factor 2 (Nrf2) expression [76]. Thus, modulating ferroptosis levels in macrophages may offer a new therapeutic direction for improving diabetic wound healing.

### Ferroptosis in fibroblasts

During wound healing, fibroblasts are responsible for synthesizing ECM and promoting repair. Studies show that fibroblast ferroptosis widely participates in wound healing. Nuclear receptor coactivator 4 (NCOA4)-mediated ferritinophagy promotes ferroptosis in skin fibroblasts induced by oxygen and glucose deprivation, leading to delayed healing of ischemic wounds [77]. In diabetic wounds, high-glucose environments impair fibroblast survival, proliferation, and migration, with multiple studies linking these defects to activated ferroptosis [49, 78, 79]. Ferrostatin-1 inhibits ferroptosis and restores these cellular functions [79]. Additionally, topical application of Ferrostatin-1 to diabetic rat wounds activates the phosphatidylinositol 3-kinase (PI3K)/protein kinase B (AKT) pathway, reduces oxidative stress and inflammatory markers, and accelerates healing [79]. Other therapeutic approaches targeting fibroblast ferroptosis have also emerged: the in vitro and in vivo experiments demonstrate that platelet-rich plasma (PRP) promotes healing of type 2 diabetic mouse ulcers by inhibiting fibroblast ferroptosis and improving functional impairment [78, 80], providing a new strategy for diabetic ulcer treatment. Furthermore, studies have found that in high-glucose-cultured human dermal fibroblasts, treatment with secretory autophagosomes derived from Human Umbilical Vein Endothelial Cells (HUVECs) can reduce endoplasmic reticulum stress-regulated free Fe²⁺ generation, while increasing fibroblast exosome release to export free Fe²⁺, thereby inhibiting ferroptosis and restoring fibroblast proliferation and migration functions. Further loading of these secretory autophagosomes into a hydrogel was shown to improve diabetic wound healing [49]. Moreover, ferroptosis is associated with pathological accumulation of senescent fibroblasts in diabetic wounds, which impairs cell motility and proliferation. Senescent fibroblasts in diabetic wounds exhibit ferroptosis resistance due to defective ferritinophagy, but upregulating the expression of the ferritin autophagy-related molecule NCOA4 increases their susceptibility to ferroptosis and improves wound healing [81]. These findings highlight that regulating fibroblast ferroptosis may represent an effective strategy for enhancing diabetic wound healing.

### Ferroptosis in endothelial cells

Among studies on ferroptosis in diabetic wound repair, endothelial cell research is the most extensive. Vascular pathology, characterized by reduced vascular and capillary density, is a major mechanism of non-healing diabetic wound [82, 83]. Vascular endothelial cells are central to angiogenesis and vascular function, regulating neovascularization, barrier homeostasis, and coagulation-fibrinolysis activity. High-glucose exposure impairs endothelial cell function, and while the exact mechanisms remain incompletely understood, recent evidence supports a role for ferroptosis. Ferrostatin-1 inhibits ferroptosis-related protein expression in endothelial cells, reduces ROS and lipid peroxidation levels, and improves cell survival and migration [79]. In addition to the effects on fibroblasts, PRP reduces lipid peroxidation and alleviates ferroptosis in endothelial cells treated with high glucose and ferroptosis inducer Erastin, improving cell regeneration and proliferation to promote diabetic ulcer healing [80]. Mechanistically, Nrf2 is widely involved in endothelial cell ferroptosis in diabetic wounds [84]. As a transcriptional coactivator, Nrf2 inhibits ferroptosis through target genes such as GPX4 and G6PD [85, 86]. For example, activation of transient receptor potential ankyrin 1 (TRPA1) induces Ca²⁺ influx, promoting calmodulin-dependent protein kinase II (CaMKII) phosphorylation, and Nrf2 nuclear translocation, which upregulates GPX4 to inhibit ferroptosis in high-glucose-exposed endothelial cells, thereby improving cell migration, proliferation, and tube formation to enhance diabetic wound healing [87]. In AGEs-treated HUVECs, resveratrol inhibits ferroptosis by upregulating Nrf2, promoting angiogenesis, and accelerating wound healing [88]. Additionally, 4-Octyl itaconate (4OI) suppresses endothelial ferroptosis by activating the Kelch-like ECH-associated protein 1 (Keap1)/Nrf2/GPX4 signaling pathway [89]. Cell-cell interactions also play an important role: neutrophil extracellular traps (NETs) released by neutrophils induce endothelial ferroptosis via inhibiting the PI3K/AKT pathway, a key factor in impaired angiogenesis in diabetic wounds [90]. Noncoding RNAs can also regulate endothelial ferroptosis: microRNA-17-92 enhances resistance to Erastin-induced ferroptosis in endothelial cells in vitro [91], while circRNA-itchy E3 ubiquitin protein ligase (circ-ITCH) activates the Nrf2 pathway by recruiting TATA-box-binding protein associated factor 15 (TAF15), inhibiting ferroptosis and improving angiogenic capacity in HUVECs [92]. Collectively, ferroptosis is closely linked to endothelial dysfunction through multiple mechanisms. Exploring new therapeutic approaches based on these mechanisms—particularly those protecting endothelial function and promoting neovascularization—holds significant promise for accelerating diabetic wound healing.

### Ferroptosis in keratinocytes

As the primary cells of the epidermis, keratinocyte ferroptosis plays a critical role in various wound injuries and skin diseases. In Stevens-Johnson syndrome and toxic epidermal necrolysis, keratinocytes exhibit imbalanced FSP1-CoQ10 antioxidant systems and increased lipid peroxidation, leading to ferroptosis and life-threatening skin adverse drug reactions characterized by keratinocyte death [93]. Moreover, ferroptosis in keratinocytes is extensively involved in the progression of psoriasis and ultraviolet-induced skin damage [94, 95]. In diabetes, accumulated AGEs represent a key inducer of keratinocyte ferroptosis. HaCaT cells exposed to AGEs exhibit reduced viability, enhanced lipid peroxidation, and increased ROS levels, leading to ferroptosis [96]. Additionally, decreased expression of Sequestosome 1 (SQSTM1) in diabetic keratinocytes impairs autophagy-lysosome-mediated degradation of ACSL4, and increasing ACSL4 expression could drive ferroptosis [48]. Exosomes derived from CoQ10-treated mesenchymal stem cells (MSCs) promote keratinocyte proliferation and migration under high-glucose conditions, inhibit ACSL4-mediated ferroptosis via delivering microRNA-548ai and microRNA-660, and accelerate skin wound healing in diabetic mice [97]. Although the specific mechanisms of keratinocyte ferroptosis in diabetic wound repair remain incompletely elucidated, targeting ferroptosis clearly improves keratinocyte function and promotes wound healing.

### Bacterial ferroptosis

In addition to repair-associated cells, bacteria play a significant role in wound healing, particularly in infection-prone diabetic wounds. Ferroptosis is generally defined as iron-dependent lipid peroxidation of PUFAs in cell membranes. While most bacterial membranes consist primarily of saturated or monounsaturated lipids, certain bacteria possess the ability to synthesize PUFAs or acquire them exogenously and incorporate them into their membranes, making ferroptosis-based antimicrobial therapy a promising approach [98–101]. Notably, inducing ferroptosis in host cells may impair their function, highlighting the value of developing bacteria-specific ferroptosis therapies. Glucose starvation-induced activation of AMP-activated protein kinase (AMPK) inhibits biosynthesis of PUFAs-containing lipids, thereby suppressing ferroptosis [102]. Leveraging this property, a research team from Sichuan University developed a novel bacteria-targeted ferroptosis bio-heterojunction (F-bio-HJ) composed of Fe₂O₃, Ti₃C₂-MXene, and glucose oxidase. F-bio-HJ triggers starvation protection in macrophages via glucose oxidase to reduce cellular ferroptosis while accelerating Fe²⁺ delivery to bacteria (both extracellularly and intracellularly) to induce bacterial ferroptosis. Animal experiments confirmed its antibacterial efficacy and wound-healing promotion in diabetic wounds infected with Staphylococcus aureus [99]. Another study developed GelMA hydrogel microneedles loaded with Fe₃O₄/MXene heterojunctions, which exhibit site-dependent ROS targeting: they scavenge extracellular ROS while releasing Fe²⁺/Fe³⁺ to enhance intracellular ROS and induce bacterial ferroptosis, demonstrating potent antibacterial activity and wound-healing promotion in diabetic infected wounds [98]. Additionally, FeCl_3_-loaded hydrogels induce ferroptosis in Pseudomonas aeruginosa, achieving antimicrobial therapy for wounds [101]. These approaches offer innovative strategies for treating infected diabetic wounds and accelerating healing.

## Therapeutic strategies targeting ferroptosis

**Fig. 1:**
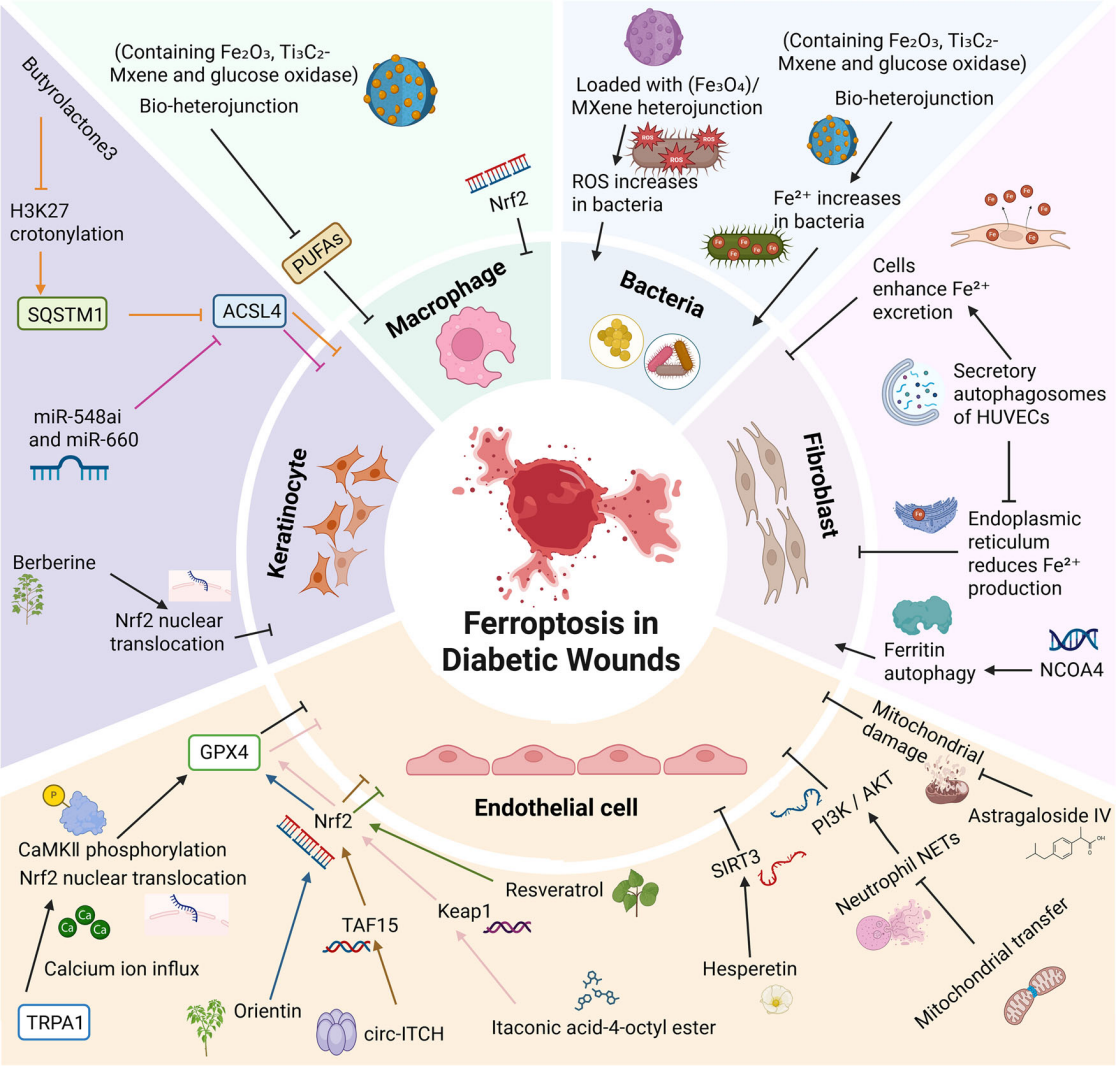
This figure illustrates four major cell types (endothelial cells, keratinocytes, macrophages, and fibroblasts) and microbial factors involved in ferroptosis of diabetic wounds. For each factor, the panel details some possible disease mechanisms and potential intervention methods. (**⊣**) indicates inhibition; (→) indicates promotion. Abbreviations in the Fig., CaMKⅡ calmodulin-dependent protein kinase II, TRPA1 transient receptor potential ankyrin 1, GPX4 glutathione peroxidase 4, Nrf2 nuclear factor-E2-related factor 2, TAF15 TATA-box-binding protein associated factor 15, Keap1 Kelch-like ECH-associated protein 1, circ-ITCH circRNA-itchy E3 ubiquitin protein ligase, SIRT3 Sirtuin 3, PI3K phosphatidylinositol 3-kinase, AKT protein kinase B, NCOA4 nuclear receptor coactivator 4, PUFAs polyunsaturated fatty acids, ACSL4 acyl-CoA synthetase long-chain family member 4, SQSTM1 Sequestosome 1.

In recent years, therapeutic strategies targeting ferroptosis have gradually emerged, particularly in the context of diabetic wound healing. Ferroptosis can delay wound repair by impairing cellular functions during the healing process. However, studies have also shown that rational utilization of ferroptosis to eliminate senescent cells and bacteria can facilitate wound management. Therefore, the role of ferroptosis should be viewed dialectically. This section systematically reviews current ferroptosis-based therapeutic strategies for diabetic wounds (Table 1 and Fig. 1). On one hand, it summarizes existing ferroptosis inhibition therapies, categorized here based on the characteristics of intervention methods: classical ferroptosis inhibitors, novel target-based ferroptosis inhibitors, natural compounds, PRP, and extracellular vesicles (EVs). On the other hand, this review innovatively compiles emerging therapies that promote diabetic wound healing by activating ferroptosis. These innovative approaches target key regulatory nodes of ferroptosis to construct a multimodal intervention framework for diabetic wounds, demonstrating high translational medicine value and precision therapy potential.

**Table 1:** Therapeutic Strategies and Targets for Ferroptosis in Diabetic Wounds.

Intervention	Cell Model	Animal Model	Potential Mechanism and Targets	Regulation of Ferroptosis	Reference
Ferrostatin-1	Macrophages cultured with high glucose	Full-thickness skin defect in diabetic rats	Promote Nrf2 expression	Inhibition	[76]
Secretory autophagosomes of HUVECs (loaded in GelMA hydrogel)	Fibroblasts cultured with high glucose	Full-thickness skin defect in diabetic mice	Reduce endoplasmic reticulum stress-regulated free Fe²⁺ production and increase fibroblast exosome release to expel free Fe²⁺	Inhibition	[49]
NCOA4 gene regulation	Senescent fibroblasts induced by high glucose	Full-thickness skin defect in diabetic mice	Upregulate NCOA4 in senescent fibroblasts and promote ferritin autophagy	Promotion	[81]
Ferrostatin-1	Vascular endothelial cells and fibroblasts cultured with high glucose and/or RSL3	Full-thickness skin defect in diabetic rats	/	Inhibition	[79]
Cinnamaldehyde, a specific TRPA1 agonist (loaded in MMP-9-responsive hydrogel)	Vascular endothelial cells cultured with high glucose	Full-thickness skin defect in diabetic mice	Activate TRPA1, induce Ca²⁺ influx to promote CaMKⅡ phosphorylation and Nrf2 nuclear translocation, upregulate GPX4	Inhibition	[87]
Ferrostatin-1	Vascular endothelial cells treated with AGEs	Full-thickness skin defect in diabetic rats	Promote Nrf2 expression	Inhibition	[88]
Hesperetin	Vascular endothelial cells induced by Erastin	Full-thickness skin defect in diabetic rats	Activate SIRT3	Inhibition	[104]
Orientin, a natural flavonoid glycoside	Vascular endothelial cells cultured with high glucose	Full-thickness skin defect in diabetic mice	Activate the Nrf2/GPX4 pathway	Inhibition	[84]
Platelet-rich plasma	Vascular endothelial cells and fibroblasts cultured with high glucose or RSL3	Full-thickness skin defect in diabetic rats	/	Inhibition	[78]
Platelet-rich plasma	Vascular endothelial cells and fibroblasts induced by high glucose and/or Erastin	Skin ulcer wound in diabetic rats	/	Inhibition	[80]
Extracellular vesicles secreted by mesenchymal stem cells	Vascular endothelial cells induced by NETs	Full-thickness skin defect in diabetic mice	Inhibit neutrophil NETs formation by transferring functional mitochondria, thereby activating the PI3K/AKT pathway	Inhibition	[90]
Exosomes derived from bone marrow mesenchymal stem cells overexpressing circ-ITCH	Vascular endothelial cells cultured with high glucose	Skin ulcer wound in diabetic mice	circ-ITCH recruits TAF15 protein to activate the Nrf2 signaling pathway	Inhibition	[92]
ROS-responsive hydrogel loaded with endothelial progenitor cell exosomes and astragaloside IV	Endothelial progenitor cells cultured with high glucose	Full-thickness skin defect in diabetic rats	Inhibit mitochondrial damage	Inhibition	[108]
Itaconic acid 4-octyl ester (loaded in exosomes and encapsulated in Alg-DA/GelMA hydrogel)	Vascular endothelial cells cultured with high glucose	Full-thickness skin defect in diabetic mice	Activate the Keap1/Nrf2/GPX4 pathway	Inhibition	[89]
Butyrolactone 3 (histone acetyltransferase inhibitor)	Keratinocytes cultured with high glucose	Full-thickness skin defect in diabetic rats	Block high glucose-induced H3K27 crotonylation, enhance SQSTM1 expression, promote autophagy and reduce ACSL4 expression	Inhibition	[48]
Berberine	Keratinocytes induced by AGEs	Normal skin of diabetic mice	Promote Nrf2 nuclear translocation	Inhibition	[96]
Exosomes derived from CoQ10-treated umbilical cord mesenchymal stem cells	Keratinocytes cultured with high glucose	Full-thickness skin defect in diabetic mice	Deliver miR-548ai and miR-660 to inhibit ACSL4	Inhibition	[97]
GelMA hydrogel loaded with (Fe₃O₄)/MXene heterojunction	Staphylococcus aureus, Escherichia coli	Full-thickness skin defect in diabetic rats infected with *Staphylococcus aureus*	Upregulate intracellular ROS in bacteria	Promotion	[98]
Bacteria-targeted bio-heterojunction (comprising Fe₂O₃, Ti₃C₂-MXene, and glucose oxidase)	Staphylococcus aureus, Escherichia coli, and E. coli-infected macrophages	Full-thickness skin defect in diabetic rats infected with *Staphylococcus aureus*	Macrophages: Glucose oxidase triggers macrophage starvation protection and inhibits PUFA synthesis; Bacteria: Accelerate Fe²⁺ uptake	Macrophages: Inhibition Bacteria: Promotion	[99]

### Therapies based on ferroptosis inhibition

#### Classical ferroptosis inhibitors

With the deepening of ferroptosis research, many classical ferroptosis inhibitors have been identified, including Ferrostatin-1, deferoxamine, Liproxstatin-1, vitamin E, etc. [103]. Among them, Ferrostatin-1 was the first radical-trapping antioxidant reported to block ferroptosis and has been widely used as a reference compound in research over the past decade [19]. In diabetic wound repair, Ferrostatin-1 demonstrates promising effects. It inhibits ferroptosis in vascular endothelial cells and fibroblasts cultured under high glucose and/or RSL3 conditions while improving cellular functions. Additionally, it suppresses macrophage ferroptosis and reduces inflammation by upregulating Nrf2 expression in high-glucose-treated macrophages, collectively promoting the healing of full-thickness skin defect wounds in diabetic rats [76, 79]. However, current research on ferroptosis-related inhibitors remains in the preclinical stage [79].

#### Novel target-based ferroptosis inhibitors

For therapeutic strategies against ferroptosis-related diseases, gene regulation and targeted drug development have become frontier directions. With the identification of key ferroptosis targets (e.g., TRPA1, H3K27 crotonylation), precise interventions using novel small-molecule drugs and biological agents show potential. For example, the TRPA1-specific agonist cinnamaldehyde significantly inhibits ferroptosis in cellular and animal models by activating CaMKII phosphorylation and Nrf2 nuclear translocation [87]. The histone acetyltransferase inhibitor Butyrolactone 3 blocks high-glucose-induced H3K27 crotonylation, activates autophagy, and suppresses keratinocyte ferroptosis [48]. Furthermore, combining CRISPR gene editing technology for directional regulation of key targets may provide new solutions for diabetic wound therapy.

#### Natural compounds

Beyond classical inhibitors, certain natural compounds have been shown to suppress ferroptosis in diabetic wound cells. Resveratrol improves ferroptosis and cellular functions in AGEs-treated vascular endothelial cells by upregulating Nrf2 expression, demonstrating wound-healing promotion in animal models [88]. In Erastin-induced endothelial ferroptosis, hesperetin exerts protective effects by activating Sirtuin 3 (SIRT3) [104]. Berberine alleviates AGEs-induced keratinocyte ferroptosis and functional impairment via promoting Nrf2 nuclear translocation [96]. The natural flavonoid orientin improves high-glucose-induced endothelial ferroptosis and cellular functions through the Nrf2/GPX4 pathway, accelerating healing of full-thickness skin defects in diabetic mice [84].

#### Platelet-rich plasma (PRP)

PRP is a plasma derivative characterized by a 3-7-fold higher platelet concentration than whole blood, achieved via centrifugation [105]. It contains rich growth factors and has a significant curative effect in promoting tissue repair, which is applied in orthopedics, plastic surgery, neurosurgery, dentistry, and dermatology. A randomized controlled clinical trial reported PRP’s potential for repairing DFUs [106]. Mechanistic studies clarify that its reparative effects on diabetic wounds likely occur by inhibiting ferroptosis in vascular endothelial cells and fibroblasts, thereby improving cellular functions [78, 80].

#### Extracellular vesicles (EVs)

EVs are membrane-bound bioactive particles secreted by cells, mediating intercellular communication through transferring proteins, nucleic acids, and lipids to regulate physiological/pathological processes in recipient cells. MSC-derived EVs have gained research attention due to their high biocompatibility, low immunogenicity, and avoidance of live-cell therapy risks. Recent studies show EVs inhibit ferroptosis through multiple pathways to improve diabetic wound healing. MSC-EVs suppress neutrophil NETosis by transferring functional mitochondria, thereby alleviating endothelial ferroptosis [90]. Engineered modifications, such as exosomes overexpressing circ-ITCH or loaded with CoQ10, deliver functional circRNA or microRNA to high-glucose-damaged endothelial cells or keratinocytes, regulating ferroptosis and promoting repair [92, 97]. Secretory autophagosomes (a type of EV) from HUVECs inhibit ferroptosis in high-glucose-treated fibroblasts by reducing free Fe²⁺ levels [49]. These findings provide new strategies for EV-based anti-ferroptosis therapy.

### Therapies based on ferroptosis activation

In diabetic wounds, cellular dysfunction or bacterial colonization promotes recruitment and retention of immune cells, leading to prolonged inflammation that creates an optimal environment for cellular senescence [107]. The senescence-associated secretory phenotype (SASP) of senescent cells can induce neighboring cell senescence, disrupting the wound repair microenvironment. Studies show senescent fibroblasts in diabetic wounds exhibit resistance to ferroptosis, a potential driver of their pathological accumulation. Thus, intervention with the ferroptosis activator Erastin to promote senescent fibroblast ferroptosis may represent an effective therapeutic strategy [81].

Bacterial colonization in wounds further exacerbates inflammation and delays healing. Therefore, bacterial clearance via effective interventions holds therapeutic potential. With advancements in materials science, targeted delivery of iron ions to bacteria to enhance oxidative stress enables specific bacterial elimination, while adjusting material components can protect host cells from ferroptosis [98, 99].

Overall, multiple therapeutic approaches targeting ferroptosis have been developed. These treatments not only demonstrate diverse development but also exhibit potential for multidimensional combined use. They can independently intervene in key ferroptosis pathways through single mechanisms and, more importantly, construct multimodal collaborative treatment systems (such as combined interventions of small-molecule drugs/target gene regulation/exosomes). Meanwhile, to further enhance the convenience of wound therapy and achieve controlled drug release, hydrogel-based (e.g., hyaluronic acid, alginate, and gelatin) delivery systems for drugs or exosomes can be established, providing an integrated “intervention-delivery” solution for precise regulation of ferroptosis in diabetic wounds [49, 87, 89, 98, 108].

## Conclusion

As a novel type of RCD, ferroptosis exhibits significant biological significance during diabetic wound healing. Studies have demonstrated that ferroptosis is closely associated with diabetes-related chronic inflammation, oxidative stress, and cellular damage, all of which collectively influence wound healing. In this review, we systematically summarized the mechanisms underlying delayed healing of diabetic wounds and research progress on the roles of ferroptosis in this process. Additionally, we elaborated on ferroptosis in different wound repair cells (macrophages, fibroblasts, endothelial cells, keratinocytes) and bacteria in infected diabetic wounds, as well as their roles and potential mechanisms in diabetic wound repair. Finally, we reviewed current therapeutic strategies targeting ferroptosis in diabetic wounds, which have shown promising effects in in vitro and in vivo studies. However, clinical translation still faces challenges, such as administration routes, dosage optimization, and toxicity assessment, which require further investigation. Future research should delve deeper into the specific regulatory mechanisms and signaling pathways of ferroptosis, clarify its exact role in diabetic wound healing, and enhance our understanding of delayed healing mechanisms to provide more potential therapeutic strategies.
